# Death of Sir Morrell Mackenzie

**Published:** 1892-03

**Authors:** 


### DEATH OF SIR MORRELL MACKENZIE.

Sir Morrell MacKenzie, of world-renowned fame, died in London on February 3, of tuberculosis, after a short illness. This distinguished physician was born in Leytonstone, in Essex, in 1837, and was educated at the London Hospital Medical College and in Paris and Vienna. He founded the hospital for diseases of the throat in Golden Square, London, in 1863. In the same year he obtained the Jacksonian prize from the Royal College of Surgeons for his essay on diseases of the larynx. He was soon afterward elected assistant physician to the London Hospital, becoming in due course full physician, and was appointed lecturer on diseases of the throat, an appointment which he held to the time of his death. He was corresponding member of the Imperial Royal Society of Physicians of Vienna, and of the Medical Society of Prague, and an honorary fellow of the American Laryngological Association.

Dr. MacKenzie was the author of numerous publications on laryngological subjects, and in particular of a systematic treatise in two volumes on "Diseases of the Throat and Nose," which is acknowledged to be a standard work, having been translated into French and German, and having a very large circulation in England and America. Dr MacKenzie was also the author of monographs on diphtheria and hay fever, and of an article on "Specialism in Medicine," which appeared in the Fortnightly Review in 1885, and which excited considerable attention. Dr MacKenzie was in attendance on Frederick III, German Emperor, during the latter's illness and was knighted in 1887. He published in 1888 "The Fatal Illness of Frederick the Noble." At the close of that year he resigned his connection with the College of Physicians. In 1889 he contributed to the Contemporary Review, some essays entitled "The Voice in Singing and Speaking."

Sir Morrell MacKenzie had suffered from bronchitis and asthma, following his recent attack of influenza. His illness, however, it was considered, had taken a favorable course. He was attended by his brother. His death was quite sudden. Only his wife was present when it occurred.—*Medical Fortnightly.*

